# Exploring views and experiences of the general public’s adoption of digital technologies for healthy lifestyle in Singapore: a qualitative study

**DOI:** 10.3389/fpubh.2023.1227146

**Published:** 2023-09-18

**Authors:** Kumarasan Roystonn, P. V. AshaRani, Fiona Devi, Peizhi Wang, Yunjue Zhang, Anitha Jeyagurunathan, Edimansyah Abdin, Lorainne Tudor Car, Siow Ann Chong, Mythily Subramaniam

**Affiliations:** ^1^Research Division, Institute of Mental Health, Singapore, Singapore; ^2^Department of Primary Care and Public Health, School of Public Health, Imperial College London, London, United Kingdom; ^3^Lee Kong Chian School of Medicine, Nanyang Technological University, Singapore, Singapore; ^4^Saw Swee Hock School of Public Health, National University of Singapore, Singapore, Singapore

**Keywords:** digital technology, healthy lifestyle, technology adoption, ethical considerations, digital public health, qualitative research

## Abstract

**Objective:**

Little is known about the general adult population’s adoption of digital technology to support healthy lifestyle, especially when they are expected to take greater personal responsibility for managing their health and well-being today. The current qualitative study intended to gain an in-depth understanding of determinants of digital technology adoption for healthy lifestyle among community-dwelling adults in Singapore.

**Design:**

A qualitative study design, with thematic framework analysis was applied to develop themes from the data.

**Setting:**

Semi-structured individual interviews were conducted with participants either face-to-face or online through a videoconferencing platform.

**Participants:**

14 women and 16 men from the general population who were between the ages of 22 and 71 years.

**Results:**

Three major themes were developed: (1) digitally disempowered (2) safety and perceived risks and harm; (3) cultural values and drives. Adoption of technology among the general population is needs-driven, and contingent on individual, technological and other cross-cultural contextual factors.

**Conclusion:**

Our findings highlight there is no one solution which fits all individuals, emphasizing the challenges of catering to diverse groups to reduce barriers to adoption of digital technologies for healthy lifestyle. Digital guidance and training, as well as social influences, can motivate technological adoption in the population. However, technical problems as well as data security and privacy concerns should first be adequately addressed. This study provides rich cross-cultural insights and informs policy-making due to its alignment with government public health initiatives to promote healthy lifestyle.

## Introduction

According to the World Health Organization, non-communicable diseases have caused more than 41 million deaths worldwide each year (1), and the risk of developing these diseases are decisively affected by lifestyle choices (2, 3). Southeast Asia faces an epidemic of these chronic preventable diseases, now responsible for more than 60% of deaths in the region (4, 5). Physical inactivity, unhealthy diet, poor sleeping and other lifestyle behaviors are strongly associated with the development of major non-communicable diseases such as cancer, heart disease, stroke, and diabetes (5, 6). In addition to premature mortality, the associated morbidity of these modifiable risk factors including direct and indirect economic costs, exerts a substantial burden on societies and healthcare systems (6, 7). For example, physical inactivity was estimated to globally cost health-care systems US$53.8 billion, with US$13.7 billion in productivity losses due to premature deaths, and was responsible for 13.4 million disability-adjusted life-years (DALYs) worldwide (8). A recent study estimated that the national healthcare spending related to modifiable lifestyle behaviors amounted to US$730 billion in the US (9). In Singapore, healthcare costs increased from S$11.1 billion in 2019 to S$15.2 billion in 2020 (10), and is expected to increase to S$59 billion by 2030 (11). Healthcare is clearly undergoing a paradigm shift; from traditional healthcare treatment towards a person-centered management of health and healthier behaviors across many world regions and in Singapore to decelerate the overwhelming burden on health care systems (12, 13). Despite the great strides made in clinical care to identify individuals with known risk factors and prescribing timely interventions to lower the risk of disease development, the persistent burden of disease today suggests a much-needed emphasis on primary prevention of disease through health promotion (14). A general conclusion is that reducing modifiable dietary and lifestyle risk factors could prevent most cases of major non-communicable diseases among high-income populations. Active and healthy lifestyles may confer benefits for multiple health outcomes related to reduction in all-cause mortality rates and improvement in mental well-being (5). These findings are profoundly important, because they indicate that these diseases are not inevitable consequences of a modern society. Furthermore, low rates of these diseases can be attained without expensive medical treatment and facilities. Population-wide primary prevention targeted at encouraging health promoting lifestyle habits should thus be the overarching priority for the response to this global crisis. In recent years, the Ministry of Health (MOH) in Singapore has launched national health campaigns for getting the population to engage in healthy lifestyle behaviors; the largest in 2016 is known as, *War on Diabetes (WoD)* campaign (15). The WoD campaign comprised efforts to promote a healthy lifestyle among the general population in Singapore, which were aimed at associated modifiable risk factors including obesity, physical inactivity, and unhealthy diet. Yet, there is suboptimal adherence to active healthy lifestyle behaviors in the general population (16). The 2019–20 National Population Health Survey in Singapore revealed that between 2013 and 2020, the prevalence of obesity has been exponentially escalating from 8.6% to 10.5% and that of overweight (including obesity) in adults has drastically increased from 34.3% to 39.1% (17, 18). National nutrition surveys in Singapore suggest that overall, consumption patterns appear to be shifting modestly toward healthier options. Between 2010 and 2018, saturated fats intake among Singaporean adults (18–69 years) was slightly lower from 38% to 36% (19). The level of confinement and other severe restrictions implemented during the coronavirus pandemic may also have had a negative influence on active and healthy lifestyle behaviors (20).

Although there have been strategic shifts in national efforts to enable and empower individuals to live out a healthy lifestyle (e.g., WoD campaign) (13, 15), more needs to be done. The national population data suggest that besides intensifying existing public education campaigns and programs, novel approaches are needed to transform the promotion of health and prevention of disease in the general population. Digital technologies are able to better promote and sustain positive lifestyle habits (21). From a public health perspective, one of the most powerful levers for influencing population health lie today in digital technological innovations that make healthy living convenient and an accessible choice (22). Prior studies have demonstrated in Western populations, the use of digital innovations to encourage and increase healthy behaviors (physical activity, diet, mood, and good sleep quality) implemented with various smart tools (e.g., wearables/smart watches, *m*health apps, nutrition apps, fitness tracking) (23). Digital technologies can enable individuals to be active participants in their health maintenance, enabling people to manage their health and make better health and lifestyle related decisions (24, 25); and may be key to tackling the current and post-pandemic challenges on how to empower individuals to engage in healthier personal lifestyle choices (21, 26). Other research also suggest increasingly higher acceptance rates for the use of technology as a healthy behavior accompaniment, through digital innovations, which may be an efficient approach to foster active and healthy lifestyles (26). Access to such technology is increasingly available around the globe, with global internet penetration rates exceeding 90% in most developed nations (27). Indeed, in Singapore, internet penetration is as high as 92% and over 90% of all adults own a smart phone. A wide spectrum of players have begun leveraging digital technologies to nudge consumers to greater participation in healthy lifestyle promoting behaviors (28) —not only public healthcare incumbents like the government, but also private entrants such as insurance conglomerates and health consumer-technology giants. One such national movement in Singapore is ActiveSG (29); complimentary for all Singapore citizens and permanent residents to promote a healthy lifestyle through sports and sporting activities virtual or otherwise. Through this nationwide movement, physical activity and nutrition programs or courses are promoted to Singaporeans across all age groups. While available as a website, ActiveSG users can also use a mobile app to facilitate participation in physical or virtual healthy lifestyle activities (30). Another example is the Healthy 365 program introduced by the Health Promotion Board (HPB) of Singapore, which gamifies wellness by awarding redeemable health points on an app for health-promoting lifestyle practices (31). More recently, HPB expanded on this with *LumiHealth*, encouraging additional healthy lifestyle activities for smartwatch users (32).

While it is encouraging that there is a rapid growth in the number and sophistication of digital innovations for active lifestyles, it is only worthwhile if these are accepted by both the young and old, and used to improve their health outcomes. However, several researchers have found that unlike the younger generation, most older adults may be digitally estranged (33, 34). Other behavioral research highlights potential frustrations with new digital technologies, concerns about privacy, and lack of support, which may likely make individuals doubt their ability to learn and adapt, and leave them unmotivated to even try the technology (35). However, the wealth of research on the use of digital technologies focused on healthy lifestyle activities are centered on Western populations.

The Technology Acceptance Model (TAM) provides a framework for understanding the adoption of technologies (36). This model structures technology acceptance on the basis of two main perceptions: namely, usefulness (the benefit from using the technology) and ease of use. Simply, consumers are more likely to adopt a new technology that is considered usable, desirable, and beneficial. TAM has since been successfully applied to other domains including healthcare (37, 38). The uptake of digital health tools and applications has also been evaluated with the TAM to good effect in other qualitative research (39, 40).

Taking into consideration the emerging evidence for digital innovations as one of the promising solutions which potentially allow easy, personalized, and accessible means to improve the well-being of individuals, we felt that it would be meaningful to examine personal experiences surrounding the determinants of digital technology adoption for promoting active and healthy lifestyle behaviors in the general population of Singapore.

## Materials and methods

### Study design and setting

A qualitative design was undertaken in the study to explore individual experiences with digital technology to promote healthy lifestyle. This study was part of a larger nationwide study that examined the knowledge, attitudes, and protective practices toward diabetes among the public in Singapore (41). The study comprised a quantitative survey, followed by a qualitative phase, to explore the barriers and facilitators of a healthy lifestyle in Singapore. The study methodology has been published in an earlier article (41). A disproportionate stratified sampling design (by age group and ethnicity) was used, where the 3 main ethnic groups (Chinese, Malays, and Indians) and 4 age groups (18 to 34 years, 35 to 49 years, 50 to 64 years, and 65 years and above) were sampled in equivalent independent proportions of about 30% and 20%, respectively. The participants for the qualitative study were recruited from among those who participated in the quantitative survey (41) and had provided written consent for re-contact for research. Briefly, eligible participants: (1) were Singapore citizens or permanent residents; (2) aged ≥21 years; (3) could speak either English, Chinese, Malay, or Tamil, and; (4) had no formal diagnosis for diabetes. Initially, participants were stratified according to age, gender, and ethnicity, and randomly selected with an online randomization software for recruitment into the qualitative phase. Subsequently, demographics of the sample were reviewed and subsequent invitations were targeted to ensure maximum variation sampling (42), with a relatively even spread across gender, age groups, ethnicities, and languages to obtain a wide representation of views across Singapore.

Written informed consent was obtained from all participants, and ethical approval for the study was granted by the relevant institutional review board, the National Healthcare Group Domain Specific Review Board (DSRB ref.: 2019/00926). This study is reported in accordance with the Consolidated criteria for Reporting Qualitative research guidelines (43).

### Public and patient involvement

Patients were not involved in the design, recruitment or conduct of the study.

### Interviews

The study period (from August 2020 to March 2021) coincided with the rapidly developing Covid-19 pandemic situation and therefore, interviews were conducted either in person or via the video conferencing platform Zoom, depending on the participant’s preference. A semi-structured interview guide (see Supplementary material) aimed to explore participants’ perspectives on healthy lifestyle; the barriers and enablers; technology for healthy lifestyle; and, programs and initiatives related to healthy lifestyle in Singapore. The main themes in the interview guide were explored with broadly open-ended questions, and prompts (e.g., “*Can you please tell me a little bit more about that?,”* “*Could you give me an example of that?”*) were used if necessary. At times, the interviews required a ‘two-way process’ (44), where interviewers also shared information about themselves and their families, which in turn drew out richness and depth in the personal accounts of participants and their experiences.

Data collection and analysis happened concurrently, allowing emergent themes to inform ongoing data collection. The team decided to end data collection when saturation was assumed to have been reasonably attained with no new themes arising from the data. Data were analyzed first from the English-language interviews before commencing with the other language (Chinese, Malay, and Tamil) interviews. This was to ensure that we had reached thematic saturation with data collection and to simultaneously observe and analyze the other language interviews for the emergence of new themes. A total of 30 interviews were conducted by experienced qualitative researchers from the study team; 20 interviews were in English, while four were in Chinese, and three were conducted in Malay and Tamil respectively. Interviews were audio recorded, and transcribed verbatim by an external provider of transcription services. These were then checked for accuracy by researchers in the study team.

### Data analysis

Data analysis was facilitated by NVivo V.11. We relied on qualitative description (45, 46) for the study design because we wanted to generate a rich and straightforward description of participant experiences and perceptions that would inform policy (47). Using the Framework analytic method (48, 49) we took a combined approach to analysis, enabling themes to be developed both inductively from the accounts of our participants and deductively from existing literature (45). Framework analysis was considered to be a better choice than thematic analysis, because it emphasizes how both *a priori* issues and emergent data driven themes should guide the development of the analytic framework (50); this was something that suited the aims of our present study, in so far as we had certain pre-defined areas we wished to explore, but also wanted to remain open to discovering the unexpected. Regular team discussions facilitated our critical exploration and discussion of participant responses, and agreement on recurring themes.

Members of our research team (AR, FD, WP, ZY, AJ, MS, and KR) first thoroughly read and re-read each transcript, and listened back to the audio-recorded interviews familiarizing themselves with the contents of the transcripts. We found this familiarization process essential in cases where the researcher analyzing the data had not been present during the interview (48). The team then independently coded the data, which involved line-by-line analysis of the data and identification of elements that appeared important to the research questions.

Next, the researchers independently developed initial themes by further refining codes and adapting, merging and sorting them into a preliminary structure representing themes and subthemes. The researchers then met to discuss and review emerging categories and ideas to construct an initial analytical framework. Themes and subthemes were reviewed multiple times to ensure external heterogeneity and internal homogeneity. On reaching a consensus, a codebook was constructed which described each code, the inclusion and exclusion criteria, and exemplars from the transcripts to assist with reliable code application.

We applied this final analytical framework, the codebook, to each transcript using NVivo. We divided the transcripts among the researchers and imported them into NVivo ready for indexing. The semi-structured interviews were the unit of analysis. We then systematically went through each transcript, highlighting each meaningful passage of text and selecting and attaching an appropriate code from the codebook. We then used NVivo to share our indexed transcripts, ensuring that each researcher could access the whole data set for the next stage.

Once all the data had been coded using the analytical framework, we reviewed and summarized the data in a matrix for each theme using Microsoft Excel. The matrix comprised of one row per participant and one column per code. We abstracted data from transcripts for each participant and code, summarized it using verbatim words and inserted it into the corresponding cell in the matrix. We also highlighted references to potentially interesting quotations within respective cells in the matrix.

The themes for this study were generated from the data set by reviewing the matrix and making connections within and between participant and categories. This process was influenced both by the original research objectives and by new concepts generated inductively from the data. We tried to go beyond descriptions of individual cases toward developing themes which offered possible explanations for what was happening within the data. Ideas were generated, explored and fleshed out through discussions with the lead researcher (MS) on the team. Our participants’ experiences and beliefs have been presented with minimally edited verbatims in the results section below.

## Results

### Participant characteristics

Thirty individuals from the general public participated in the study, of which 16 were male and 14 were female. The mean age of participants was 44.7 years (SD = 14.7), with ages ranging from 22 to 71 years. Forty percent identified their ethnicity as Chinese, 33.3% as Malay, 20% as Indian and 7% as Others. Majority of the participants reported being married (70%), employed (66.7%), and most had attained secondary level education or higher (86.7%). Table 1 presents the demographic details of all participants.

**Table 1:** Demographic characteristics of the participants.

Characteristics	Mean (SD) / Percentage (*n*)
Age (years)	44.7 (14.7)
Gender	
Male	53.3 (16)
Female	46.7 (14)
Ethnicity	
Chinese	40.0 (12)
Malay	33.3 (10)
Indian	20.0 (6)
Others	6.7 (2)
Marital status	
Single/Never married	23.3 (7)
Married	70.0 (21)
Divorced/Separated/Widowed	6.7 (2)
Education level	
Primary level & below	13.3 (4)
Secondary level	26.7 (8)
Diploma/Vocational or ITE/Pre-university level	26.7 (8)
University level & above	33.3 (10)
Employment status	
Employed	66.7 (20)
Unemployed	13.3 (4)
Homemaker	13.3 (4)
Student/Never employed	6.7 (2)

SD, Standard deviation; ITE, Institute of Technical Education.

### Local context of digital technology adoption for healthy lifestyle

Our participants reported that using digital technologies affected their health status and lifestyle in some way. These digital technologies promoting active and healthy lifestyle behavior were mobile applications (apps), wearable devices, social media platforms and websites. The most commonly used were mobile health apps, most times associated with a wearable fitness activity tracking device.

Most participants also shared that they used one or more types of digital technologies concurrently. Participants from ethnic minority groups (Indians and Malays) expressed a tendency to use digital technology for weight and nutrition related activities such as weight management, healthy food consumption, and nutrition or calorie information compared to the Chinese majority. While many reported downloading or accessing digital tools of their own volition, more than a third, on the other hand, also reported using digital technology infrequently or not at all after.

### Determinants of digital technology adoption for healthy lifestyle

Table 2 presents the themes and subthemes relating to determinants of technology adoption for healthy lifestyle. Three broad themes (with up to three subthemes each) were developed: (1) digitally disempowered; (2) safety and perceived risks and harm; and (3) cultural values and drives. Each main theme and subthemes will be discussed in the following paragraphs.

**Table 2:** Themes and subthemes relating to determinants of technology adoption for healthy lifestyle.

***Theme 1: Digitally disempowered***
Lack of capability
Language barriers
Poor technology design and quality
***Theme 2: Safety and perceived risks and harm***
Security and privacy
Distrust and discontentment
Threat to health
***Theme 3: Cultural values and drives***
Social norms
Peer influences
Self-directed motivation

### Theme 1: Digitally disempowered

The theme “digitally disempowered” was used to describe a small but significant group of participants who believed their access and use of digital technology was hampered by a lack of capability or language barriers. This theme also captured those who believed they were hindered by poor technological design and quality to engage with these digital technologies.

#### Lack of capability

Most participants were challenged by a lack of, or an inadequate level of specific digital skills to access and use the variety of technological innovations available. In general, participants felt a significant barrier to digital technology use was their age. Among these participants, some shared that they felt the use of digital technology for active and healthy lifestyle required significantly higher levels of digital knowledge and skills which were too complex and demanding for them to learn today.

“knowledge wise, you see if you take me at my age, I don’t have that level of scientific knowledge, knowledge to use (technology) or all of these” – Male, 35–39 years.

Instead, some felt that there was a lack of guidance and training to acquire these digital skills in order to take advantage of available digital tools.

“for those who are new to going online, they still don’t know. It may be an obstacle. Because I totally don’t know; I don’t know how to book or go see. That’s what I mean. Then, if someone teaches, I will know, and it will be easy, like that” – Female, 55–59 years.

Interestingly, few older participants were among those who reported a lack of digital skills or training. Some older adults shared how they were capable, and currently engaging with digital technology to engage in active lifestyle behaviors.

“sometimes we open to this YouTube right, has exercises; to follow healthy lifestyle kind of exercise. They show you how to do this or do that…we use YouTube a lot” – Female, 65–69 years.

#### Poor technology design and quality

Poor technology quality or otherwise ill-suited designs of digital devices and smart instruments were found to hinder the possibilities of use. Some participants felt the current quality of smart wearables was still sub-par and the ill-suited designs or poor affordances meant they were not convenient or led to unappealing personal costs and effort to have them fixed.

“My friend wears a smartwatch, but mine is spoiled, so I didn’t change it because it is too difficult…I stopped. Just like that” – Female, 55–59 years.

Other participants aired their grievances about how existing digital tools lack innovation and sophistication to circumvent what they felt were cognitively challenging experiences with digital technology promoting active and healthy lifestyle behavior. For instance, having to repeatedly look at digital screens while trying to follow physical exercises or the need for frequent playback to observe the exercises more closely in order to get them right. Many shared these experiences were too cumbersome and off-putting.

“I really used it only for a few months now then gave up… it's very difficult because you will need to watch the screen as you do the exercise. For me, it's very difficult” – Female, 35–39 years.

#### Language barriers

Participants also felt that sometimes language was a challenge to using digital technology. Several participants who were not English-literate, felt there was a severe lack of digital tools, particularly mobile apps, in their native languages.

“I mean, for us people who only speak or understand Tamil…A lot of us, who only know Tamil, we won’t know a lot of things on because of this you see” – Male, 35–39 years.

### Theme 2: Safety and perceived risks and harm

This theme, “safety and perceived risks and harm” was described by the participants as one of the important determinants for adoption of digital technology for healthy lifestyle. Our participants reported three key subthemes: security and privacy, distrust and discontentment, and perceiving threats to health.

#### Security and privacy

Security issues and a lack of trust complicated the adoption of technology particularly among consumers of technology. Many participants expressed safety concerns about the security of personal information shared with various health-promoting digital tools and platforms in order to receive personalized, authentic, and meaningful experiences. Participants felt that their fear and anticipation of consequences regarding misuse of their personal data led to their avoidance or discontinued use of the technology.

“I'm not very comfortable with the idea of sharing such personal data with technology companies….I think that's really very scary to me. Yeah, taking over our lives. And what we can or cannot do. So yeah.…because they really do steal data from their own (users)” – Male, 20–24 years.

#### Distrust and discontentment

Digital technologies associated with physical activity, sleep, mood and weight management are very popular in the general population; however, the quality of the digital technologies and health information propagated on these are hard to assess for making informed health care decisions by users.

“These are the biggest negative factors online, I feel, a lot of gimmicks, a lot of scams, a lot of these kind of people that claim to know what they're doing but they don't” – Male, 25–29 years.

Experiences of distrust of digital content also emerged from the interviews, as participants shared about how they grappled with the challenge of false, inaccurate, and misleading information in digital technology promoting healthy lifestyle.

“So if a company wants to sell its product, it can really buy off a few YouTubers, popular YouTubers and tell them to sell their products. So I think this can really sway a lot of people…Basically, spread a lot of half-truths or misinformation” – Male, 20–24 years.

A number of participants felt dissatisfied particularly with mobile health apps. While most have a free version, it tends to be limited in functionality and often inundated with advertising. Participants also shared feeling deceived by ‘premium’ apps and ‘paid’ apps. They shared how it was difficult to find suitable and effective apps to achieve their lifestyle goals.

“They are just traps to get you to spend money. So it is very difficult to like figure out which ones are legitimate and which ones are out to get your money… even for a paid app, it doesn’t really guarantee results” – Female, 25–29 years.

Some participants felt most of the digital technology for active and healthy lifestyle were primarily targeted at consumers from Western populations. Participants felt that often these digital tools were not always culturally relevant, or worse, causing physical and psychological harm to uninformed users in non-Western populations.

“I think relying on it may not be very helpful, or it can actually disadvantage you because your body is definitely very unique and different from others. So your body is probably very different from that of an average Westerner. So with Western companies telling us what we should or should not do or eat, it can be affecting us very differently” – Male, 20–24 years.

#### Threat to health

In addition to the above safety concerns, a substantial number of participants commonly described digital technology as potentially harmful to healthy living. While many acknowledged that digital technology was useful for promoting healthy living, they shared how it can also bring a lot of distractions which may jeopardize their plans to engage in healthy lifestyle behaviors.

“I think when people are just stuck on their phones non-stop, it shows that technology is really not helpful for healthy living…a lot of people will end up just watching or using it for so long and it stops us…from doing our exercise” – Female, 60–64 years.

However, several participants reported that technology use as a distraction was in fact useful in motivating them to last longer during activities such as exercise. Thus, participants felt digital distractions can be a double-edged sword.

“It’s like oh I want to watch just another episode or something on Netflix and then after that, I will go (exercise)…But then because they are distracted by the show, they run a bit more. So it is really like a double-edged sword. It distracts you but it also helps you to do more of your fitness stuff because you are distracted” – Female, 25–29 years.

### Theme 3: Cultural values and drives

The theme, “Cultural values and drives” was identified as another key determinant of technological adoption. There were three subthemes: Social norms, Peer influences, and Self-directed motivations.

#### Social norms

Traditionally, healthy lifestyle interventions have been in-person activities conducted individually or in a group. A group of participants held a keen preference for these methods over the use of digital technology, as the latter was not seen to provide an equivalent experience or beneficial one. Unlike the ‘digitally disempowered’ described earlier, whose technology adoption was predominantly hampered by accessibility issues, participants in this social group shared common values to do with the undesirability of digital technology for healthy lifestyle and an avoidance of it. In addition, security and privacy concerns were also rather common in this faction of non-adopters.

“the best way, right, is through human to human. That's the best way because like, for my sister, she didn't get proper training on apps. So, it turns out she's not getting slimmer, she's getting bigger”– Male, 30–34 years.

Many of the participants shared challenges related to the nature of communication and poor interaction through the digital environment, whereas in-person sessions were thought to reduce potential misunderstandings because they provided opportunities to clarify, ask questions, receive more accurate feedback, and enhance the experience. Additionally, participants felt it was considerably less personalized in the digital environment and had doubts about receiving quality services remotely.

“Online thing is not very good. Some people might think, “I'm not sure whether am I doing correct or not though I'm following it,”…the instructor might have a hard time telling them what is the correct posture, what you should be feeling because they are not with them” – Male, 35–39 years.

#### Peer influences

Yet, peer influences surrounding an individual were found to affect participants’ propensity to engage with digital technology for healthy lifestyle. Peer opinions carried significant influence and could affect one’s personal attitudes to various digital innovations, based on the prevailing attitudes within the social network.

“Sometimes like our friends they will send us online messages, "Sis, this or that exercise online is very good," so I will just follow from there just like that…” – Female, 65–69 years.

#### Self-directed motivation

Many participants felt that these digital technologies intentionally or inadvertently give us an edge, promoting healthy lifestyle activities. They shared how leveraging technology, such as fitness apps and online coaching platforms, provided them quick access and flexibility to take up one or more workout routines at their convenience, and adapt their fitness goals to suit a variety of fitness levels at any time.

“during the circuit breaker, I downloaded a gym exercise app, and I did some gym, some weight training at home for the weight loss. And then recently, I changed to yoga from the same app. And then I did the running app. It was the app that was from couch to 5K (laughter)…and now I'm just continuing…” – Female, 45–49 years.

Participants also tended to agree on the importance of personal motivation in order to benefit from digital technology for healthy lifestyle.

“I looked at my screen time, and my screen time was three hours on the phone. And I'm like, "Oh my god. What is this? This is such a great waste of time." …. And I said, "One hour out of that time, I could have used it for doing something probably, something useful." So then, yeah, I think it's just finding the motivation is probably the biggest obstacle” – Female, 45–49 years.

## Discussion

This study was the first attempt to investigate the challenges experienced by the general population in using digital technology promoting healthy lifestyle. The themes (*Digitally disempowered; Safety and perceived risks and harm; and Cultural values and drives*) illustrated the key determinants of digital technology adoption as perceived and experienced by an ethnically diverse sample of adults in Singapore. In this discussion, we highlight our key research findings, a local conceptual model on digital technology adoption, discuss the limitations of our study and discuss directions for future research. We discuss our findings on the experiences and perceptions of digital technology through a technology acceptance (36) lens. TAM suggests that technology adoption can be explained by two main perceptions: namely, usefulness or the benefits derived from using the technology, and the ease of use.

### Perceived utility of digital technology

Our findings suggest individuals felt there were several benefits from using digital technology for healthy lifestyle. These included descriptions of its flexibility and capacity to accommodate the rapidly changing needs of individuals, and the capability to engage and motivate users. Individuals in our study also found much value in the functions, features and content available in digital technology for fitness activity and health and nutrition information. Research has suggested that the performance expectations for digital and mobile applications have a strong correlation with behavioral intentions of technology adoption (51). Our results reaffirm one of the constructs of the TAM in that individuals who appreciate the value associated with digital technology are positively influenced in their behavioral intentions of use.

### Perceived risks of digital technology

On the other hand, our results elucidate that trust and privacy concerns directly hold significant negative effects on intentions of use and the utility of digital technology among the general public in Singapore. Our findings revealed widespread concerns about the security and privacy of personal data in these digital tools and services for healthy lifestyle. Other cross-cultural researchers have reported similar barriers to technological adoption, that is, beyond the two main constructs posited by the TAM, privacy and security concerns reduce intentions to adopt healthcare technology (52, 53).

Collectively, besides improving technological functions, features, and content, it is important to consider these perceived risks of technology use and safety concerns related to inadequate protection of data and privacy (54, 55). Since the digital field is rapidly advancing, there may be a need for a neutral regulatory body for an up-to-date evaluation of digital technology, to inform consumers about reliable digital tools with data protection and privacy regulatory adherence. Local governments could provide a central database of high-quality digital interventions and services and could potentially consider involving the community in the co-ownership and management of such resources (56). This approach may help more individuals make better informed health care decisions confidently and to protect against misinformation and potential harm, effecting greater technological adoption. In general, technology adoption research tends to focus on drivers of usage intentions such as perceived usefulness, and perceived ease of use. However, our results suggest perceived risks and harms in the context of using digital technology for healthy lifestyle is a potentially important determinant of technology adoption.

### Personal norms and peer influences

As another point of departure from the TAM, our study findings uncovered the influence of peers and personal values among the Singaporean public as a major determinant that influences adoption intentions in this population. We found that members of one’s peer network affect individuals’ adoption of digital technology, as the opinions of these social contacts matter. Consistent with literature, our local population were positively influenced to adopt technology through their social contacts and personal referents, as well as external sources, such as media (57). Accordingly, it would be crucial for national public health program developers to bear this in mind, i.e., consider targeting the social or peer influence circles surrounding select individuals directly, to improve the uptake of digital innovations for promoting healthy lifestyle.

Personal norms represent one’s perceptions of moral obligation or responsibility to perform, or not to perform a behavior (i.e., adoption of technology), beyond perceived social pressure (58). Likewise, our results illustrated a significant barrier to digital technology adoption among certain individuals who undervalued and disfavored digital technology for healthy lifestyle activities despite a largely positive societal attitude observed toward digitalization and technological adoption. This resistance toward digital innovations was seen among the same individuals who voiced strong concerns about the perceived risks associated with digital technology as discussed earlier in our article. Research suggests that the risks are construed as a subjective perception about engaging with anything digital or the Internet in itself, and invariably has a negative impact on their intentions to adopt technology. These suggest that it is important to first assess the level of digital readiness among these individuals, and further underscores the need for particular health promotion strategies to engage and incentivize people while mitigating potential threats to privacy and security to improve the uptake of digital tools for promoting a healthy lifestyle. Thus, future research in this area is urgently recommended.

### Perceived ease of use of digital technology for healthy lifestyle

The results of our study revealed that a small but significant proportion of the general population were digitally disempowered and felt they were challenged by the ease of use of digital technology for healthy lifestyle for reasons including digital skills, language limitations and the complexity of digital innovations.

### Inadequate digital skills and knowledge

According to the TAM, self-efficacy renders positive effects on the perception of usability of technology while lack of knowledge and experience negatively affects ease of use. Similarly, our findings indicate the most obvious personal barrier was issues related to digital skills and capabilities of individuals, particularly those who were among the digitally disempowered. A typical challenge for individuals, regardless of age, was that their current levels of knowledge and skills were inadequate. Other research has also discovered that the problem of a lack of digital skills has broad effects in a general population (59, 60) in terms of technological adoption. Further, our study participants were also facing other challenges, such as the lack of training and guidance, which is consistent with previous literature (61). Since 2017, a national exercise to build up basic digital skills and digital literacy has helped many individuals including older adults to embrace digital life and services including digital interventions promoting healthy lifestyle (62, 63). However, the training and voluntary support programs which had helped many to gain digital access, were discontinued or turned into digital events due to the COVID-19 health crisis; further alienating those who were already lacking basic digital skills from participating. Despite the steady rise in digital services for healthy lifestyle, support may still be lacking for certain groups of individuals in the general population and necessitate immediate attention to reduce the digital divide in the population.

### Language limitations in digital tools

One unique and significant barrier negatively affecting perceived ease of use among our participants, was found to be due to language limitations. English tends to be used as the primary ‘working language’ digitally throughout the world, with a billion others speaking it as a second language (64). This has allowed for most digital tools to be built around English as the default language, even if the coding that provides the basis for final platforms and applications are in specific computer languages. This means those who can navigate the *lingua franca* can easily access and utilize such digital technology better, to the detriment of non-English speaking, less digitally-connected individuals. Researchers have highlighted that language difficulties pose significant challenges to the adoption of digital technologies, especially among ethnic minorities who may struggle with weak language skills (65, 66). Developing user-friendly digital technology for healthy lifestyle and improving physical infrastructure and support systems for troubleshooting continue to be common problems affecting one’s technological adoption (67).

### Dealing with complexity of digital innovations

Notwithstanding, inability for the end user to troubleshoot hardware and software increases the complexity of using the digital innovations which affects their perceived ease of use and has also been linked to the perception of usefulness. On the basis of the TAM, applying Roger’s theory of the diffusion of innovations (68), current literature has confirmed innovativeness may significantly influence the intention and motivation to technology adoption, where users with high innovativeness are able to handle uncertainty and thus show greater acceptance and adoption of technology (69, 70). These findings suggest a need for constructive input from key stakeholders, namely the potential users, together with designers of digital health innovations. Some researchers have suggested that external variables including individual differences, social influences, and facilitating conditions such as technical infrastructure and support for use of the technology should be taken into account (51). A multifaceted approach is required, which can address the full range of strategic and technical issues to enhance technology adoption for healthy lifestyle. Looking at these issues together, mitigating strategies such as developing digital capabilities and social support, and improving the remote support infrastructures for technology are vital to reduce the digital divide and improve access to and adoption of technology for health promoting activities (71).

### Local conceptual model of digital technology adoption for healthy lifestyle

**Figure 1:**
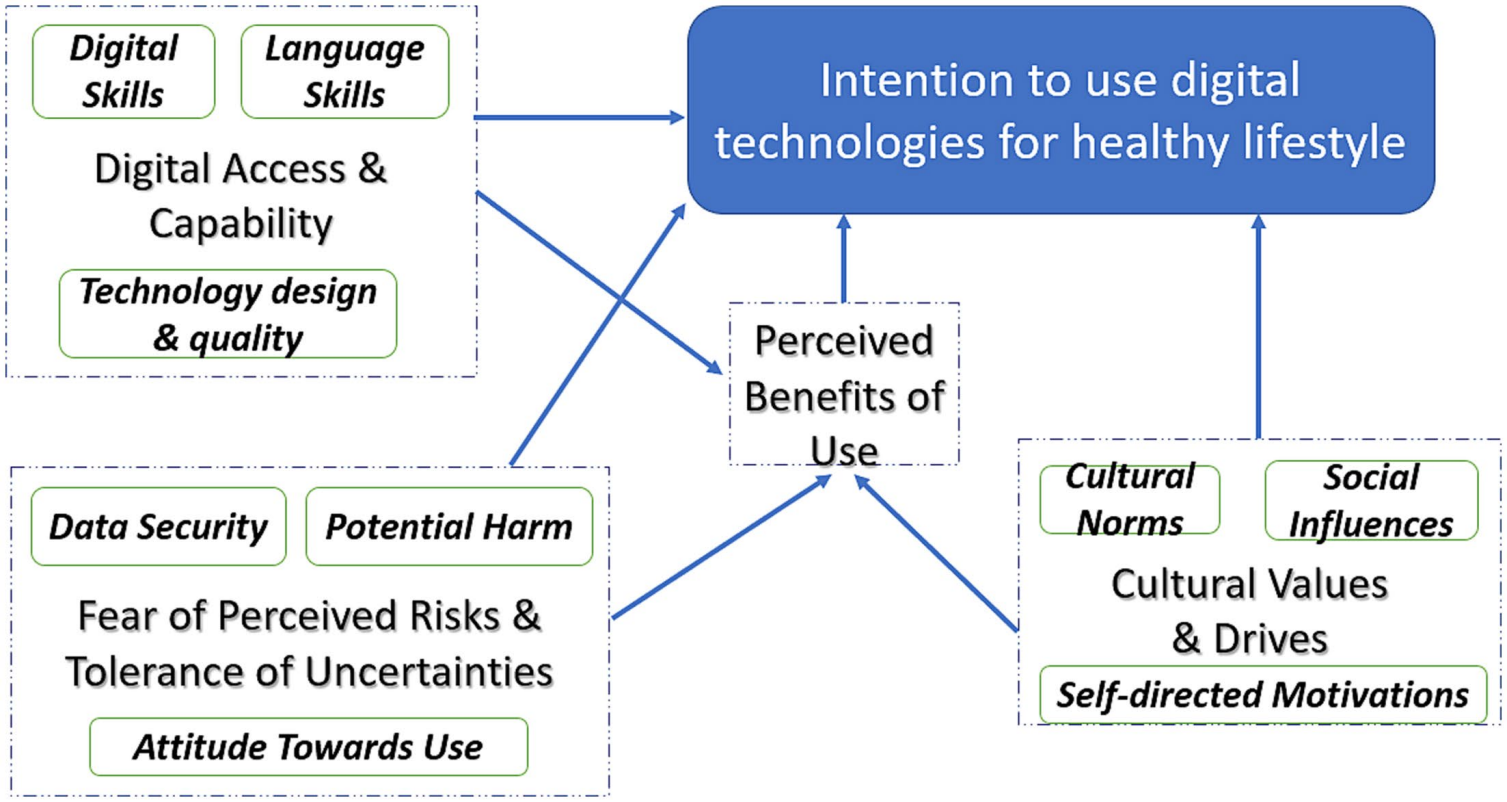
Proposed local conceptual model of digital technology adoption for healthy lifestyle.

The development of a cross-cultural model in a plural Asian society such as Singapore, is imperative to public health policy and practice; keeping abreast of the digital impacts on one’s health and health-promoting behaviors. The proposed exploratory model is architectured around the factors influencing the adoption of digital technology in the context of our rich, interview data. To this end, and to develop meaningful insights, a local conceptual model integrating unique constructs with constructs related to the technology acceptance model has been proposed (see Figure 1).

In Figure 1, *Perceived Benefits of Use*, the degree to which the consumer perceives that the technology is useful, is an original TAM construct and remains in effect. In our local context, the effortless use of the digital technology is also vital for technological acceptance and use and has been considered within *Digital Access and Capability* parameters of digital skills, language skills, and technology design and quality. Similar to the TAM, we acknowledge both perceptions of the benefits of use and the ease of access can directly and indirectly influence behavioral intentions to use digital technology for healthy lifestyle. Further, we extend the TAM by capturing two unique nuances in our model of determinants namely, “*Fear of Perceived Risks and Tolerance of Uncertainties*,” and “*Cultural Values and Drives*.” We propose that subjectively weighed perceptions of risks and tolerance of uncertainties such as security and privacy of data, as well as, trust of digital content affect attitude and intentions to use. Intuitively, we can argue that consumers will not perceive usefulness in a technology that is likely to invade their privacy or believed to cause potential harm. We argue it will affect perceived usefulness negatively and indirectly influence intentions to use. Additionally, in our local model, we consider the unique influence of social and personal norms as the other major factor that influences adoption intentions. The attitudes and beliefs of social groups and personal referents significantly influence value judgments on the utility of technology and intentions to use digital technology. This model explores the interactions and relationships among the factors emergent in our study as significant determinants of adoption of technology. These various factors interact with one another in this multi-dimensional model which underscores key opportunities and targeted strategies to intervene.

### Practical implications

Based on the notable challenges experienced by the general public, it can be argued that several key areas for development are necessary to encourage the use of digital health interventions for active and healthy lifestyle behavior. Our results suggest that one of the major problems in accessing such digital technology is related to one’s level of digital skills, although, this study identified significant challenges in other digital determinants as well. Continued efforts to improve basic digital skills and equitable digital access among underserved groups will be beneficial. Additionally, in future, it will be important to invest in information about digital health services through various channels because the opportunities and potential benefits of these services has not been disseminated widely enough to reach everyone. Increasingly, both public and private stakeholders have begun leveraging digital technologies to nudge consumers toward monitoring their health and lowering the long-term cost of care.

Initial engagement with digital tools appeared to stem in most cases from self-directed motivations. Increased health consciousness and an uptake in technology means that there is likely to be a pressing need to examine how technology can reduce barriers and help people maintain the positive behavioral changes. This study hence further demonstrates the crucial need for additional support for on-going motivation and development of habitual routines for health-promoting activity. Investing in research and development for technologies such as digital conversational agents that explicitly motivate and support effective behavior change and habit formation could be a valuable public health strategy given the potential for maximizing reach in populations who may be disproportionately utilizing healthcare resources (72). Similarly, another potential avenue for this would be creating digital resources using participatory research or citizen science, which will help to ensure that the most pertinent digital tools and features are used in a way that will enhance engagement and the likelihood of behavior change (73). Moreover, it is wise to note that traditional face-to-face services for healthy lifestyle practices will continue to be important among certain groups in the population, and should still be maintained and provided alongside digital tools and services in a possible blended type of approach.

### Study strengths and limitations

Our study has considerable strengths and few limitations. Strengths include the broad and diverse sample of participants interviewed, including males and females across age, ethnicity and language groups. Limitations of our study include the fact that our sample comprised participants who had volunteered to be contacted for this qualitative study and thus, our interviewed participants may have more positive experiences or be more willing to share their perceptions related to the topic. We interviewed participants in the midst of the rapidly developing coronavirus situation, and so it is not certain whether experiences would differ in the longer term. In addition, our sample comprised residents who lived in Singapore, spoke English, Chinese, Malay or Tamil, and had good to excellent Internet connectivity. Therefore, our findings in this study may not be transferable to those in other settings and in other countries.

### Conclusion

In conclusion, the main objective of this study is to examine the general population’s experiences and the factors influencing the adoption of digital technologies for healthy lifestyle. On the basis of TAM, this study found evidence for both perceived usefulness and the ease of use, but also contributed to new cross-cultural understandings of the phenomenon, with fear of perceived risks and cultural value and drives as potential antecedents of the adoption of digital technology. Participants appreciated the value of digital technology and mostly perceived the ease of use positively in Singapore which encourages digital technology for healthy lifestyle. However, despite efforts spearheaded by the Singapore Government, participants identified several barriers to technology adoption including a lack of digital skills, language barriers, and fear of perceived risks and harm on digital tools and platforms. On the other hand, social and peer influences emerged as a significant mechanism that can be leveraged to improve adoption of digital technology.

### Future works

While future developments should invest more in usability research and the features of novel health-promoting digital tools, a much-needed consideration is to enhance data security research as well as to communicate a better understanding of private data use to allay concerns and improve the public adoption of digital innovations promoting healthy lifestyle. Future research should also examine if there is a paradigm shift in the population of how individuals engage with digital technology for healthy lifestyle purposes. The range of reasons for use and ways in which the resident population engage with digital tools to practice healthy lifestyle behaviors highlight there is no one solution which fits all individuals, highlighting the challenges of catering to diverse groups with varying engagement with digital technology. Factors influencing intentions to use digital technology may be different in long-term participation and maintenance of behavior. The processes and determinants could be more complex and require extensive investigation, particularly in this digitally-driven, post-pandemic future. Subsequent research should reveal the rich temporal process of engagement with digital technology promoting healthy lifestyle, that could not be possible in the current study.

## Data availability statement

The datasets presented in this article are not readily available because restrictions apply to the availability of these data, which are not publicly available because of ethical and institutional regulations. Requests to access the datasets should be directed to MS, mythily@imh.com.sg.

## Ethics statement

The studies involving humans were approved by National Healthcare Group Domain Specific Review Board. The studies were conducted in accordance with the local legislation and institutional requirements. The participants provided their written informed consent to participate in this study.

## Author contributions

KR was the primary contributor to the writing of the article, conducted the interviews, and collaborated in data analysis. SC contributed to the design of the study and applied for the necessary approvals. MS contributed to the design of the study, conducted the interviews, collaborated in data analysis, and contributed to the writing of the article. PA, YZ, FD, PW, AJ, EA, and LC contributed to the writing of the article. All authors have made critical comments on the article, reviewed the article for its intellectual content, and approved the manuscript.

## Funding

This study is supported by the Singapore Ministry of Health’s National Medical Research Council under its Health Services Research Grant (NMRC/HSRG/0085/2018). The funders had no role in the design of the study, collection and interpretation of data, or preparation of the manuscript.

## Conflict of interest

The authors declare that the research was conducted in the absence of any commercial or financial relationships that could be construed as a potential conflict of interest.

## Publisher’s note

All claims expressed in this article are solely those of the authors and do not necessarily represent those of their affiliated organizations, or those of the publisher, the editors and the reviewers. Any product that may be evaluated in this article, or claim that may be made by its manufacturer, is not guaranteed or endorsed by the publisher.
